# Detection of Water pH Using Visible Near-Infrared Spectroscopy and One-Dimensional Convolutional Neural Network

**DOI:** 10.3390/s22155809

**Published:** 2022-08-03

**Authors:** Dengshan Li, Lina Li

**Affiliations:** College of Mechanical Engineering and Automation, Huaqiao University, Xiamen 361021, China; 20013080028@stu.hqu.edu.cn

**Keywords:** visible, near-infrared, pH detection, one-dimensional convolutional neural network, multivariate regression calibration

## Abstract

pH is an important parameter for water quality detection. This study proposed a novel calibration regression strategy based on a one-dimensional convolutional neural network (1D-CNN) for water pH detection using visible near-infrared (Vis-NIR) spectroscopy. Two groups of Vis-NIR spectral analysis experiments of water pH detection were employed to evaluate the performance of 1D-CNN. Two conventional multivariate regression calibration methods, including partial least squares (PLS) and least squares support vector machine (LS-SVM), were introduced for comparative analysis with 1D-CNN. The successive projections algorithm (SPA) was adopted to select the feature variables. In addition, the learning mechanism of 1D-CNN was interpreted through visual feature maps by convolutional layers. The results showed that the 1D-CNN models obtained the highest prediction accuracy based on full spectra for the two experiments. For the spectrophotometer experiment, the root mean square error of prediction (RMSEP) was 0.7925, and the determination coefficient of prediction ($R_{p}^{2}$) was 0.8515. For the grating spectrograph experiment, the RMSEP was 0.5128 and the $R_{p}^{2}$ was 0.9273. The convolutional layers could automatically preprocess the spectra and effectively extract the spectra features. Compared with the traditional regression methods, 1D-CNN does not need complex spectra pretreatment and variable selection. Therefore, 1D-CNN is a promising regression approach, with higher prediction accuracy and better modeling convenience for rapid water pH detection using Vis-NIR spectroscopy.

## 1. Introduction

Water is the basic resource for human survival and ecosystem evolution. Water safety is an issue that cannot be overlooked and that directly affects the earth′s ecological environment and human survival. However, with the development of society and economics, industrial waste is emerging as a principal source of water contamination. Water quality parameters, such as pH, dissolved oxygen, turbidity, sediments, chloride ions, potassium ions, and so on, characterize the quality of the water environment. pH is one of the important water quality parameters, as it involves the water′s natural phenomena, chemical changes, and production process [1]. Therefore, research on the rapid detection of water pH is significant.

Traditionally, the determination of water pH mainly adopts the glass electrode method [2]. The measurements of this method are accurate, but the process is cumbersome and needs the pH buffers to calibrate. Visible near-infrared (Vis-NIR) spectroscopy is a fast, non-destructive and qualitative analysis technique which has been widely used in the rapid detection of chemical composition [3,4,5]. In the Vis-NIR region, the absorption of the spectrum mainly comes from the frequency doubling and combination band of hydrogen-containing groups (such as O-H, C-H, and N-H) [6]. Vis-NIR spectroscopy combined with chemometrics is one of the promising techniques for rapid water pH detection. However, since the prediction accuracy is not satisfactory, there are few reports about the use of this technology to detect the water pH value [7].

High-accuracy and effective multivariate regression calibration models play a key role in Vis-NIR spectral analysis. A lot of multivariate calibration algorithms have been developed to build the relationship between the Vis-NIR spectra and the target attributes, including partial least squares (PLS), least squares support vector machine (LS-SVM), artificial neural network (ANN), and extreme learning machine (ELM) [8,9,10], etc. Among these methods, the PLS (linear) and LS-SVM (nonlinear) are the most commonly used multivariate calibration methods [11]. The high dimension and high complexity are the main characteristics of modern spectroscopy. The spectra data contains a large number of uninformative variables and noise, which is the challenge of spectra analysis. In order to reduce the dimension of spectra and improve the prediction accuracy, a large amount of characteristic wavelength variable selection methods were proposed [12]. However, many variable selection algorithms are of low reproducibility and have the risk of overfitting, such as competitive adaptive reweighted sampling (CARS), genetic algorithm (GA), and variable combination population analysis (VCPA) [13].

In recent years, convolutional neural networks (CNN) have provided new insights for spectra analysis. CNN is a representative deep learning algorithm which was initially used to solve the spectra classification problems. Recent studies have found that CNN is feasible in spectra multivariate regression calibration, and the prediction performance of CNN is better than PLS. Cui et al. (2018) proposed a unified CNN structure used for multivariate regression, and the results indicated that the CNN models achieved the highest prediction accuracy [14]. Li et al. (2022) explored the potential of using the short-wave infrared (SWIR) hyperspectral imaging (HSI) technique combined with a one-dimensional convolutional neural network (1D-CNN) for predicting the SSC of dried Hami jujube, and the results indicated that the prediction accuracy of 1D-CNN was satisfactory [11]. Mishra et al. (2022) presented a strategy of 1D-CNN modeling for the multi-response prediction for spectral data of fresh fruit, they found that the prediction performance of 1D-CNN is better than PLS [15]. Moreover, CNN modeling has no need for complex spectra pretreatment and variable selection, since the CNN with convolutional layers can automatically extract the features of spectra [13]. Thus, CNN is expected to be a widely used multivariate calibration technology in Vis-NIR spectroscopy analysis.

Although the CNN model has outperformed the traditional calibration methods, it is hard to explain how CNN extracts efficient information from the input spectra and how CNN establishes the relationship between the spectral and the objective attribute. For linear regression strategies such as principal component regression (PCR) and PLS, the absolute value of regression coefficients indicates the importance of the corresponding wavelength variables. The nonlinear regression methods, such as LS-SVM, ELM, and CNN, have more complex calculation processes and structures, and it is difficult to explain the relevance between the spectral data and the attribute of interest. The CNN model is a black box due to the complex network structure and multilayer operations. Interpreting the learning mechanism of CNN is critical for further application. In the field of spectra classification, CNN’s visual explanation method has already been reported. Fukuhara et al. (2019) proposed a feature visualization method that calculates important regions in the spectra from weights in pooling and fully-connected layers [16]. This approach focuses on explaining the characteristic variables extraction but ignores the explanation of how the convolutional layers process the spectra and learn the spectra features. Zhang et al. (2020) and Ng et al. (2020) visualized the feature maps of convolutional layers to explain how the CNN model processes the spectra and extracts the feature variables [13,17]. This method explains the contribution of each variable to the classification results and interprets how the convolutional layers process the spectra and learn the spectra features. However, in the field of spectra regression, there are few reports about interpreting the CNN regression model.

Therefore, a novel calibration regression strategy based on 1D-CNN for water pH detection is proposed in this study. Two experiments of water pH value detection using Vis-NIR spectral analysis are adopted to evaluate the performance of 1D-CNN. PLS and LS-SVM are introduced for comparative analysis with 1D-CNN. The main goal of this study are the following: (1) explore the potential of using Vis-NIR spectroscopy for water pH detection; (2) establish and optimize traditional models (PLS and LS-SVM) through spectra pretreatment methods and characteristic wavelength variables algorithms; (3) construct the 1D-CNN architectures and optimize the parameters of 1D-CNN for modeling to predict water pH; (4) visualize the feature maps by convolutional layers and interpret the internal feature representations of 1D-CNN; (5) compare the prediction performance of 1D-CNN and traditional multiple regression algorithms, the model prediction accuracy, and convenience of modeling are discussed.

## 2. Materials and Methods

### 2.1. Experiment

In this study, two independent experiments were conducted to evaluate the effectiveness and applicability of the 1D-CNN regression method for water pH detection using Vis-NIR spectroscopy. In these two experiments, we adopted two Vis-NIR spectrometers with different spectroscopic principles to measure the absorbance of samples.

Sample preparation: Randomly added the 0.1 mol/L HCl solution or 0.1 mol/L NaOH solution into distilled water to prepare samples with different pH values.

Reference pH measurement: Use the pH meter (Shanghai Lichen-BX Instrument Co. Ltd., Shanghai, China) to measure the reference pH of each sample. The measurement accuracy of the pH meter is ±0.01 pH unit.

#### 2.1.1. Spectrophotometer Experiment: Detecting the pH Value of Distilled Water with a Spectrophotometer

Sample description: A total of 34 samples were used in this experiment. The reference pH range was 3.19~10.63, and the average value and standard deviation were 6.15 and 2.08, respectively.

**Figure 1:**
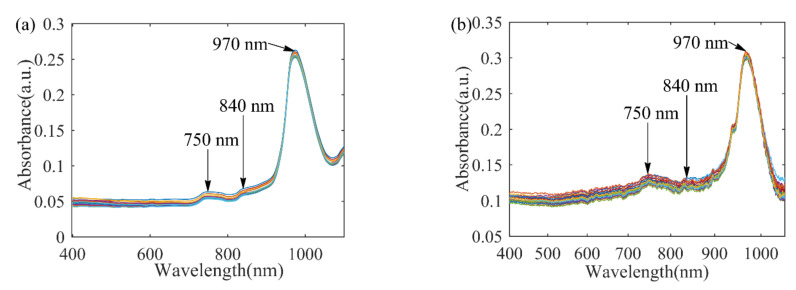
Original Vis-NIR spectra of distilled water samples: (**a**) spectrophotometer experiment; (**b**) grating spectrograph experiment.

Spectra acquisition: A spectrophotometer (V-1800PC, Shanghai Mapada Instrument Co. Ltd., Shanghai, China) with a resolution of 2 nm was employed to measure the Vis-NIR spectra in the range of 400~1100 nm. Each sample was placed in a 10 mm quartz cell. Adopted transmission measurements to obtain the spectra and record the absorbance as the spectra data. The original Vis-NIR spectrum of the 34 distilled water samples are shown in Figure 1a.

Sample grouping: In order to ensure that the multivariate calibration model has a good generalization ability, the distilled water samples were divided into a calibration set (24 samples) and a prediction set (eight samples) by the descending sorting method [18]. The calibration set is used for modeling, and the prediction set is adapted to test the performance of the multivariate calibration model.

#### 2.1.2. Grating Spectrograph Experiment: Detecting the pH Value of Distilled Water with a Grating Spectrograph

Sample description: A total of 32 samples were used in this experiment. The reference pH range was 1.64~9.31, and the average value and standard deviation were 6.27 and 2.03, respectively.

Spectra acquisition: The spectra acquisition system consists of a light source (HL-2000-LL), two transmission fibers, and a grating spectrograph (FLAME-T-XR1-RS, Ocean Optics Inc., Orlando, FL, USA). The wavelength range was 400~1049 nm and the spectra resolution were 2860 pixels. Each sample was placed in a 10 mm quartz cell. Spectrometer parameters include an integration time of 34 ms, smoothing 3, and average times 200. Adopted transmission measurements to obtain the spectral and record the absorbance value as the spectra data. The original Vis-NIR spectrum of the 32 distilled water samples is shown in Figure 1b.

Sample grouping: The 32 distilled water samples were partitioned into two parts by the descending sorting method, including a calibration set (24 samples) and a prediction set (8 samples).

#### 2.1.3. Spectral Reference Characteristics

As shown in Figure 1, the key chemical features appear near the ~970 nm, which is related to the third overtones of O-H bonds in water [19]. The small mounds near ~840 nm and ~750 nm, which can be correlated to 2*v*_1_ + *v*_2_ + *v*_3_ and 3*v*_1_ + *v*_3_ combination transition, respectively, where *v*_1_ is the symmetric O-H stretch, *v*_2_ is the O-H bending mode, and *v*_3_ is the antisymmetric O-H stretch [20].

### 2.2. Traditional Modeling Methods

#### 2.2.1. Spectral Preprocessing Methods

The spectra data is easily disturbed from instrumental drifts, measurement modality, sample state, and other environmental factors in the acquisition process [21]. Three commonly used preprocessing strategies are applied in this study, including Savitzky-Golay (S-G) smoothing, standard normal variate (SNV), and Z-score normalization. The S-G smoothing with a second-order polynomial and seven smoothing points (SG (2,7)) was used to improve the spectral smoothness and reduce noise interference. The purpose of SNV is to remove scatter noise [22]. The spectra data by Z-score normalization conforms to the standard normal distribution [23].

#### 2.2.2. Modeling Algorithms

Two traditional calibration algorithms, PLS and LS-SVM, are adopted for comparative analysis with 1D-CNN. These two calibration algorithms are briefly introduced as follows.

(1) Partial least squares regression is one of the most commonly used calibration methods, which establishes a linear connection between the spectra data matrix (***x***) and the target attributes (*y*). PLS extract uncorrelated principal components (PCs) from the spectra to construct the calibration models. For more details about PLS, please refer to reference [24]. In this study, according to the root mean square error of cross-validation (RMSECV), we chose the optimum number of PCs (nPCs) [18].
(2) Least squares support vector machine is a commonly-used machine learning algorithm which exhibits high prediction accuracy in addressing linear and nonlinear problems [25]. LS-SVM employs a kernel function to transform the original spectra data into a high-dimensional space. Then support vectors are obtained by a set of linear equations. For more details about LS-SVM, please refer to reference [8]. The prediction results of LS-SVM can be expressed as Equation (1):
  (1) $$\hat{y}=\overset{n}{\underset{i=1}{\sum }}\alpha_{i}\cdot K\;\left(x,\;x_{i}\right)+b$$
  where ***α****_i_* correspond to the Lagrange multiplier called support vector, *K* (***x***, ***x****_i_*) represents the kernel function, ***x*** refers to the spectra data in the prediction set, and *b* is the bias.

Before building the LS-SVM model, three crucial factors were considered, including the optimal input features, kernel function, and optimal model parameters [26]. Firstly, the raw or the preprocessed spectra was adopted as the input dataset. Secondly, the radial basis function (RBF) was selected as the kernel function. The RBF kernel can reduce the computational complexity and has an excellent performance in dealing with nonlinear problems [25]. Finally, two model parameters need to be optimized, including the regularization parameter (*γ*) and the parameter (*δ*^2^) of RBF.

This research employed the particle swarm optimization (PSO) algorithm to search the optimal *γ* and *δ*^2^. The range of *γ* and *δ*^2^ within 10^−2^ to 10^6^ [25,26]. PSO algorithm to find the optimal solution by iteration, according to different problems, the corresponding fitness function is used to evaluate the quality of the solution [27]. Compared with other optimization methods, the advantage of PSO is easy to realize, and has high solution accuracy and calculation speed. For the PSO, parameters include the population size of particle swarm (20), learning factors *c*_1_ = *c*_2_ = 1.5, inertia weight *w_max_* = 1.2 and *w_min_* = 0.8, and the velocity *v_max_* = −*v_min_* = 20. The fitness function of the PSO algorithm is the root mean square error of calibration (RMSEC). After 200 iterations, the optimal parameters of LS-SVM were obtained.

#### 2.2.3. Successive Projection Algorithm (SPA)

Characteristic wavelength variables selection can eliminate the noise and uninformative variables in the original spectra. Models established based on the small numbers of characteristic wavelength variables are less costly and easy to interpret.

The successive projection algorithm is a forward variable selection method [7]. SPA has a good performance in solving the collinearity problems of spectra data. The objective of SPA is to select a subset of variables whose information content is at least redundant [28]. Firstly, SPA projects the variable onto other variables, and the variable of the largest projection vector is selected as the characteristic variable. It then incorporates a new one at each iteration until the best *m* variables are selected. It is worth noting that the *m* value is not more than the number of samples in the calibration set. More details about SPA can be found at reference [29].

All spectra pretreatment and traditional models were implemented using MATLAB R2016b (The MathWorks Inc., Natick, MA, USA).

### 2.3. One-Dimensional Convolutional Neural Network (1D-CNN)

#### 2.3.1. Data Augmentation and Spectral Preprocessing

In this study, the number of distilled water samples is small for 1D-CNN modeling. The raw spectra data were augmented using the data augmentation algorithm proposed by Bjerrum et al. to avoid the overfitting phenomenon in the training process and improve the robustness of the 1D-CNN model [30]. This method adds random offset, multiplication, and slope into the original dataset. The offset was varied −0.1~0.1 times the standard deviation of the calibration set. The multiplication was done with 0.9~1.1 times the standard deviation of the calibration set. In addition, the slope was uniformly randomly adjusted between 0.95~1.05.

For the spectrophotometer experiment, the raw spectra were first processed by Z-score normalization. Then, each sample in the calibration set was done 10 times data augmentation and appended to the dataset. After data augmentation, the calibration dataset has a total of 264 spectra.

For the grating spectrograph experiment, the raw spectra were first processed by Z-score normalization. Then, each sample in the calibration set was done 10 times data augmentation and appended to the dataset. After data augmentation, the calibration set has a total of 264 spectra. Finally, the calibration set and prediction set were subjected to SNV preprocess.

#### 2.3.2. 1D-CNN Architecture

The CNN basic architecture generally includes input layer, convolutional layer, pooling layer, activation function layer, flatten layer, fully connected layer, and output layer, etc. The main function of convolutional layers is to extract the characteristics of input spectra [13,31]. Take the first convolutional layer as an example, the convolutional layer with *N* same size filters. After the convolutional layer, the input spectra are transformed to *N* feature maps. However, the convolution is a linear operation. In order to implement nonlinearity transformation in the network, the feature maps are passed to an activation function layer [31]. The generally used activation function includes sigmoid function, rectified linear units (ReLU), and exponential linear units (ELU). The main purpose of the pooling layer is to reduce the dimensional of convolutional layer feature maps, which helps increase the calculation speed and prevent overfitting. The fully connected layer is a multi-layer perceptron, and each neuron in this layer is connected to all the elements in the previous layer [18].

**Figure 2:**
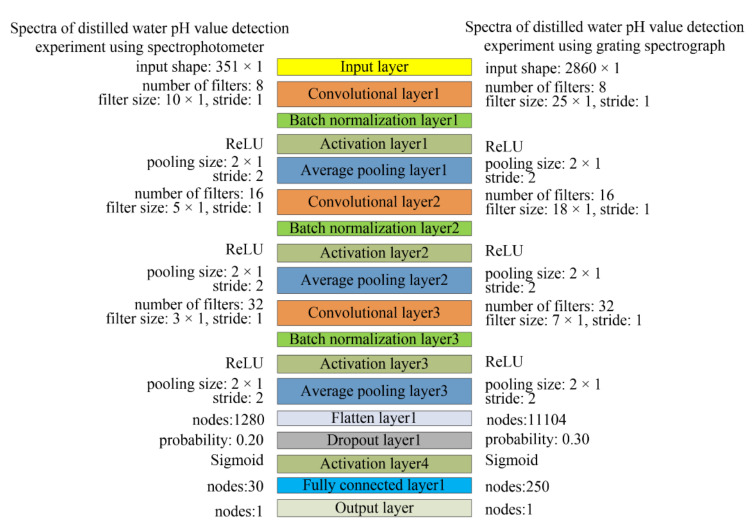
Architecture of 1D-CNN models.

Inspired by the classic CNN network structures of LeNet-5 [18], as shown in Figure 2, this study constructed a 1D-CNN framework to predict the water pH value. The 1D-CNN model consists of an input layer, three convolutional layers, three batch normalization layers, four activation function layers, three average pooling layers, a flatten layer, a dropout layer, a fully connected layer, and an output layer. The pooling size is 2 × 1 and the stride is 2. The purpose of the batch normalization layer is to standardize the data of each mini-batch and normalize the output into a standard normal distribution, which is an effective regularization strategy [18]. In order to prevent overfitting and improve the calculation speed, a batch normalization layer was added after each convolutional layer. We added a dropout layer after the flatten layer. The dropout layer randomly drops out nodes to further reduce overfitting. The output layer predicts the pH value. It is worth noting that the output layer is a fully connected dense layer with one node.

For the spectrophotometer experiment, the input of the 1D-CNN model is one-dimensional spectra data which have a total of 351 wavelength variables, so the shape of the input layer is 351 × 1. The number of filters in the three convolutional layers is 8, 16, and 32, respectively. The ReLU is the activation function added between the batch normalization layer and the pooling layer. The sigmoid is the activation function added after the dropout layer.

For the grating spectrograph experiment, the shape of input layer is 2860 × 1. The number of filters in the three convolutional layers is 8, 16, and 32, respectively. The ReLU is the activation function added between the batch normalization layer and pooling layer, and the sigmoid is the activation function added after the dropout layer.

The hyperparameters of 1D-CNN models (filter size, node number of fully connected layer, and dropout probability) were optimized according to the number of samples, the number of convolutional layers, and the spectral dimension [13]. The optimal results of hyperparameters are shown in Figure 2.

#### 2.3.3. Training of 1D-CNN

In the 1D-CNN model training process, mean squared error (MSE) was employed as the loss function and calculated in Equation (2) [11].

(2) $$loss=MSE=\frac{\sum_{i=1}^{n}{\left({\hat{y}}_{i}-y_{i}\right)}^{2}}{n}$$

where *n* is the number of water samples in the calibration set, ${\hat{y}}_{i}$ and $y_{i}$ are the predicted pH and reference pH of the *i*th water samples, respectively. The initial learning rate was 0.001. We adopted the adaptive moment estimation (Adam) optimizer to minimize the MSE through the gradient descent algorithm.

**Figure 3:**
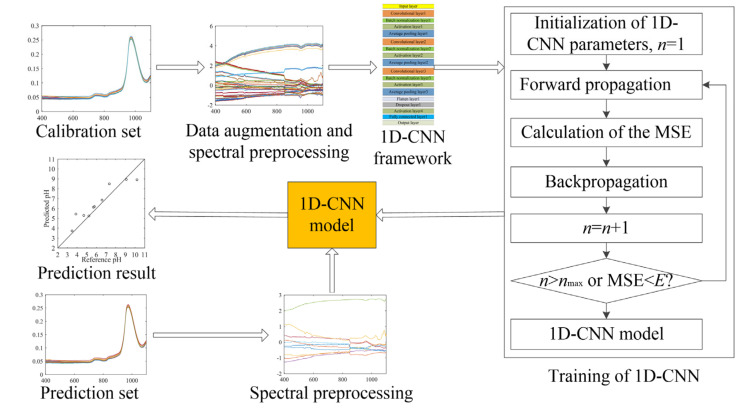
The flowchart of water pH detection using Vis-NIR spectral analysis based on 1D-CNN. In this figure, *n* is the training epochs and *E* is the expected value of MSE.

The flowchart of water pH detection using Vis-NIR spectral analysis based on 1D-CNN is shown in Figure 3. The 1D-CNN model training process was as follows:

(1) Data augmentation and spectral preprocessing. Before 1D-CNN training, in order to improve the prediction accuracy and prevent overfitting. As previously described, after Z-score preprocess, the calibration set was augmented 10 times using the data augmentation method.
(2) The parameters of 1D-CNN, including all layer weight and biases, were initialized randomly.
(3) Forward propagation. The spectra in the calibration set as the input data of the 1D-CNN finally acquired the predicted pH values from the output layer.
(4) Calculate the MSE value between the predicted and the reference pH values by equation (2).
(5) Backpropagation. Calculate the error gradient of the output layer, and use the backpropagation algorithm to calculate the error gradient of each weight. Then, use the gradient descent algorithm to update the weight value in each layer. The purpose of this step is to optimize the weight of 1D-CNN to minimize the MSE [32].
(6) Go to step (3) until the training epochs reach the maximum number of training epochs or the MSE value is less than the set value.

For the spectrophotometer experiment, the batch size was set to 24. The model has been trained for 180 epochs to make sure it was fully trained. For the grating spectrograph experiment, the batch size was set to 24, and the model has been trained for 80 epochs.

All 1D-CNN models were implemented using the Python (3.7.1) programming language and TensorFlow (1.14.0).

### 2.4. Criteria for Model Evaluation

The performance of the calibration models is evaluated according to the values of the root mean square error (RMSE) of calibration (RMSEC), prediction (RMSEP), and coefficient (*R*^2^) of determination calibration ($R_{c}^{2}$) and prediction ($R_{p}^{2}$) [11,33]. Generally, the lower value of RMSE and the closer the *R*^2^ is to 1 indicate that the prediction result from the calibration model is more reliable and the calibration model has a better-predicted performance [11].

### 2.5. Outlier Recognition

Outliers contained in the calibration set may have a significant effect on the calibration result [34]. Particularly for the 1D-CNN model, the MSE loss function is more sensitive to outliers [35,36]. In order to reduce the influence of outliers in the calibration model training process, leave-one-out cross-validation (LOOCV) with the 3σ criterion was employed for outlier recognition [37]. This algorithm firstly constructs the PLS model, then adopts the leave-one-out manner to calculate the standard deviation σ(*i*) of the prediction error *e*(*i*) for the *i*th sample. If the absolute value of *e*(*i*) is larger than the absolute value of 3σ(*i*), the *i*th sample is an outlier sample and should be eliminated. For more details about LOOCV with the 3σ criterion, please refer to reference [37].

**Figure 4:**
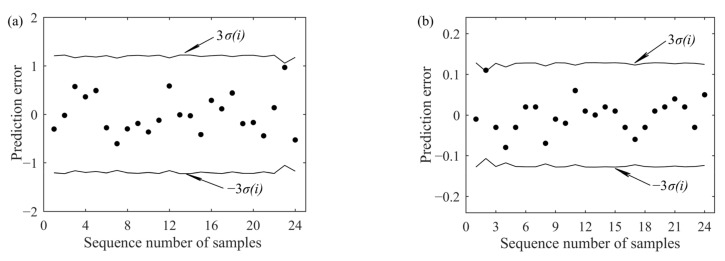
Results of LOOCV with the 3σ criterion: (**a**) spectrophotometer experiment; (**b**) grating spectrograph experiment.

Figure 4a,b show the results of LOOCV with the 3σ criterion for two groups of water pH detection experiments. The results show that there are no outliers in the calibration set.

## 3. Results and Discussion

### 3.1. Prediction Results Using Traditional Modeling Methods

To compare the effects of different preprocessing methods on the spectra analysis, PLS and LS-SVM calibration models based on different preprocesses were established.

For the spectrophotometer experiment, the prediction results are given in Table 1. Under the PLS, for the raw spectra, the RMSEP is 1.1381, and the $R_{p}^{2}$ is 0.6942. For other preprocessing methods, the prediction results show a decrease to some degree. Under the LS-SVM, for the raw spectra, the RMSEP is 1.0295, and the $R_{p}^{2}$ is 0.7495. With the comparison of the prediction results for different preprocessing methods, the SG (2,7) smoothing preprocess achieved the best prediction accuracy, the RMSEP is 1.0290, and the $R_{p}^{2}$ is 0.7498.

For the grating spectrograph experiment, the prediction results are given in Table 2. Under the PLS, for the raw spectra, the RMSEP is 1.1496 and the $R_{p}^{2}$ is 0.6569. Compared with the raw spectra, the prediction accuracy of the model based on SG (2,7) smoothing shows a slight increase. While the prediction results of the models based on other preprocessing methods show a decrease to some degree. Under the LS-SVM, for the raw spectra, the RMSEP is 1.1991, and the $R_{p}^{2}$ is 0.6025. Compared with the prediction results based on different preprocessing methods, the Z-score normalization acquired the best prediction accuracy, the RMSEP is 1.0228, and the $R_{p}^{2}$ is 0.7108.

### 3.2. Characteristic Wavelength Selection and Validation

**Figure 5:**
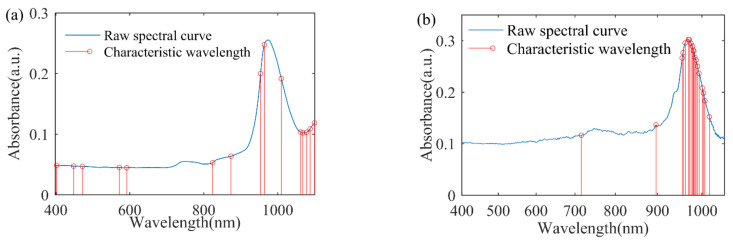
The distribution of wavelength variables selected by SPA: (**a**) spectrophotometer experiment; (**b**) grating spectrograph experiment.

SPA was employed to select the characteristic wavelength variables and simplify the calibration models. For the spectrophotometer experiment, after extensive experimentation, using the raw spectra to select characteristic wavelength variables can acquire the best prediction accuracy. A total of 15 characteristic wavelength variables were selected. Figure 5a shows the distribution of the selected wavelength variables. The characteristic wavelength variables are 402 nm, 448 nm, 472 nm, 572 nm, 592 nm, 824 nm, 874 nm, 954 nm, 964 nm, 1010 nm, 1062 nm, 1068 nm, 1078 nm, 1088 nm, and 1100 nm.

For the grating spectrograph experiment, before characteristic wavelength selection, the spectra were preprocessed by the Z-score normalization and SNV. A total of 19 characteristic wavelength variables were selected. Figure 5b shows the distribution of the selected wavelength variables. The characteristic wavelength variables are 716 nm, 896 nm, and the band of 956~994 nm and 1004~1017 nm.

To evaluate the effectiveness of characteristic wavelength variables, SPA-PLS and SPA-LSSVM models were established by taking these characteristic wavelength variables as the input data matrix. For the spectrophotometer experiment, the prediction results are given in Table 3. Under the SPA-PLS, the RMSEP is 1.0209 and the $R_{p}^{2}$ is 0.7539. Under the SPA-LS-SVM, the RMSEP is 1.1286 and the $R_{p}^{2}$ is 0.6990.

For the grating spectrograph experiment, the prediction results are given in Table 4. Under the SPA-PLS, the RMSEP is 0.5737 and the $R_{p}^{2}$ is 0.9145. Under the SPA-LS-SVM, the RMSEP is 0.5211 and the $R_{p}^{2}$ is 0.9249.

### 3.3. Prediction Results of 1D-CNN

In order to estimate the effectiveness of 1D-CNN for water pH detection using Vis-NIR spectroscopy, 1D-CNN is introduced to build the calibration model. Table 5 lists the prediction results of 1D-CNN models. For the spectrophotometer experiment, the RMSEP is 0.7925 and the $R_{p}^{2}$ is 0.8515. For the grating spectrograph experiment, the RMSEP is 0.5128, and the $R_{p}^{2}$ is 0.9273.

### 3.4. Interpreting the Feature Representations of Convolutional Layers

To further understand how the 1D-CNN extracts intricate features hierarchically, we took one sample from the prediction set to visualize the feature maps of each convolutional layer [13,17].

**Figure 6:**
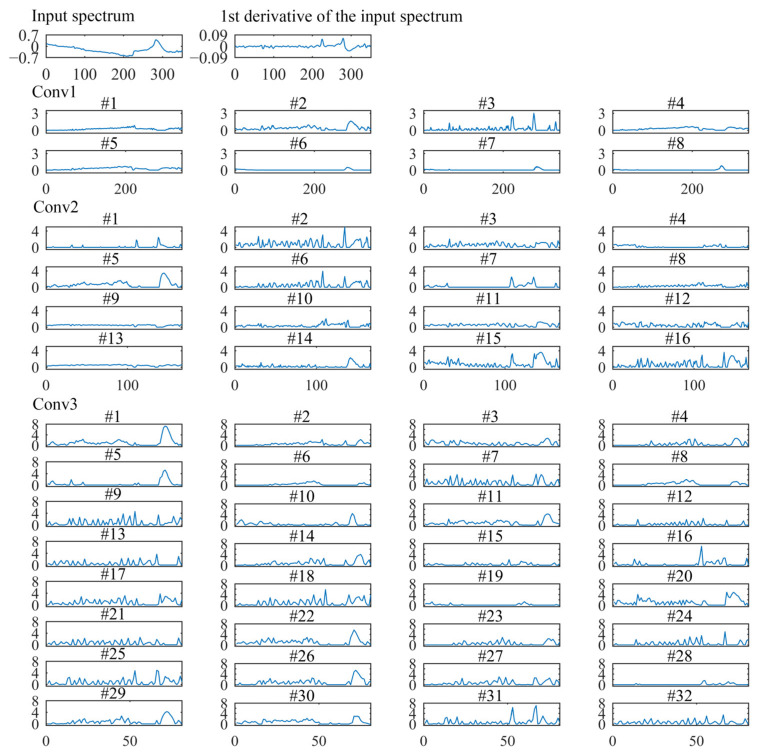
Visualization of the input spectral, the first derivative of the input spectral, and the feature maps of each convolutional layer for the spectrophotometer experiment.

For the spectrophotometer experiment, Figure 6 shows the input spectral and feature maps of each convolutional layer. The first convolutional layer convolutes the input spectra to generate eight feature maps. Then, these eight feature maps are used as the input of different channels for the second convolutional layer to generate 16 feature maps. Similarly, the third convolutional layer generates 32 feature maps. Since the spatial invariance of the 1D-CNN, the feature map corresponds to the input spectral. Therefore, visualization of feature maps is helpful to understand the data transformation by convolutional layers [13].

The first convolutional layer (Conv1) mainly acts for the spectra preprocess and learns the shape characteristics of the spectra [17,30]. As shown in Figure 6, the effect of many filters (#1, #2, #3, #4, and #5) are similar to the commonly spectral preprocessing methods. The effect of the #3 filter is similar to the first derivative preprocessing. Three feature maps (filters #6, #7, and #8) show nearly zero activations in the whole spectrum, except for high responses in the band of near ~970 nm. The zero activations indicate that these filters are not sensitive to the input wavelength variables.

The second convolutional layer (Conv2) enhances the response of spectra peaks to extract the informative wavelength variables [30]. As shown in Figure 6, half the number of filters (#1, #2, #5, #6, #7, #14, #15, and #16) have high activations on variables between 960~980 nm, and the theoretical peak around 970 nm is related to the third overtones of O-H bonds in water [19]. Seven filters (#1, #2, #6, #7, #10, #15, #16) have high responses on variables between 840~860 nm, which around the O-H weak absorption bands [20]. The uninformative variables are reduced to zero.

Deeper in the network, the third convolutional layer (Conv3) becomes more complex. As shown in Figure 6, the feature maps show a stable increased activation on the spectra peaks. The higher activation value indicates more contribution to the final calibration regression result. On the contrary, the zero activations do not influence the final calibration regression result [13].

**Figure 7:**
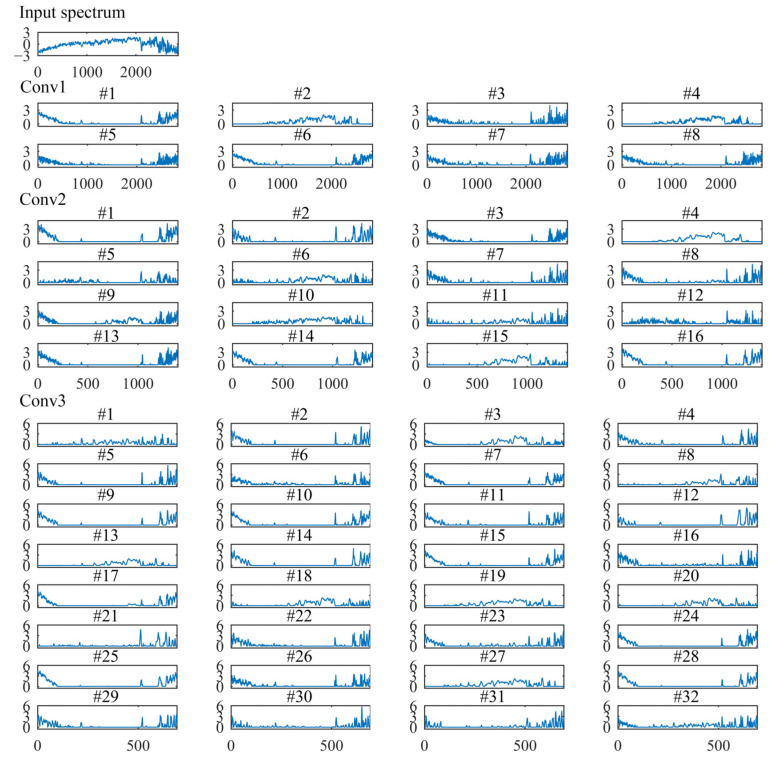
Visualization of the input spectral and feature maps of each convolutional layer for the grating spectrograph experiment.

For the grating spectrograph experiment, Figure 7 shows the input spectral and feature maps of each convolutional layer. In the first convolutional layer (Conv1), the effect of many filters is preprocessing the input spectra [13]. Six filters (#1, #3, #5, #6, #7, and #8) have high responses on the band of 400~500 nm and 960~1049 nm.

The second convolutional layer (Conv2) works something like feature variable selection. The Conv2 has enhanced the informative peaks on a few of the characteristic wavelength variables, while the uninformative wavelength variables were almost zero activations. Most filters have high activations on wavelength variables on 400~500 nm, 890~900 nm, and 960~1049 nm. In the third convolutional layer (Conv3), a stable increased activation on the wavelength band at 400~500 nm, 890~900 nm, and 960~1049 nm was noted. The results indicate that these wavelength variables contribute more greatly to the final calibration regression result.

### 3.5. Calibration Performance Comparisons Discussion of the Multivariate Calibration Models

#### 3.5.1. Discussion of Model Prediction Accuracy

**Figure 8:**
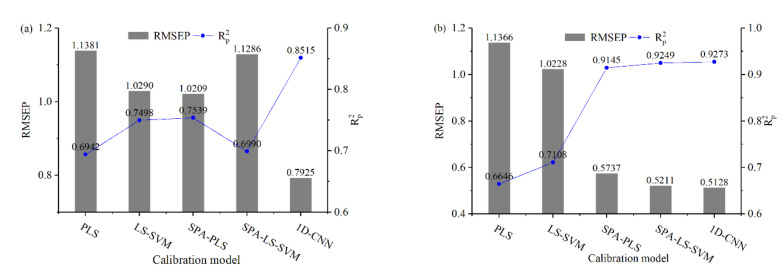
The best prediction accuracy of different calibration models: (**a**) spectrophotometer experiment; (**b**) grating spectrograph experiment.

**Figure 9:**
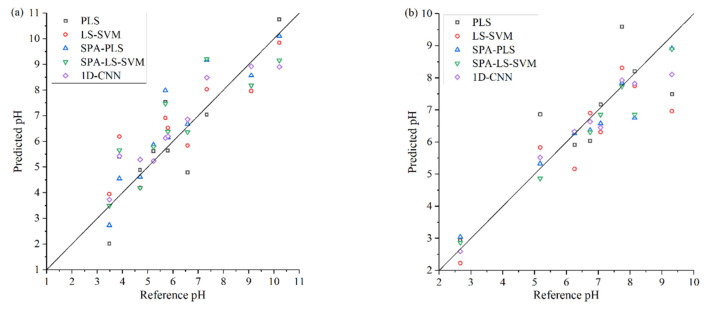
Scatter plots of reference pH and the predicted pH obtained by different regression models: (**a**) spectrophotometer experiment; (**b**) grating spectrograph experiment. The solid line in each figure represents the ideal regression line.

The best prediction results and scatter plots of predicted results and reference pH values obtained by different calibration models are shown in Figure 8 and Figure 9, respectively. 

As shown in Figure 8a, for the spectrophotometer experiment, the 1D-CNN model achieved the best prediction performance, the RMSEP is 0.7925 and the $R_{p}^{2}$ is 0.8515. Compared with the SPA-PLS model, the RMSEP is reduced by 22.37%, and the $R_{p}^{2}$ is increased by 12.95%. As shown in Figure 9a, the 1D-CNN model shows that more sample points are closed to the ideal regression line, which indicates that the prediction performance of 1D-CNN is better than the other four traditional regression models [11,38].

As shown in Figure 8b, for the grating spectrograph experiment, both the SPA-PLS, SPA-LS-SVM, and 1D-CNN models acquired good prediction accuracy. The 1D-CNN model achieved the best prediction performance, the RMSEP is 0.5128, and the $R_{p}^{2}$ is 0.9273. As shown in Figure 9b, both the sample points of SPA-PLS, SPA-LS-SVM, and 1D-CNN models are closed to the solid line, which indicates that these three models could predict the water pH accurately.

Compared with the prediction accuracy of different calibration models for the water pH detection experiments, the results show that the prediction performance of the 1D-CNN models is better than that of other traditional calibration models. The root cause of better prediction performance of 1D-CNN is the well-trained convolutional layers that can extract the spectra features more effectively and accurately [11,18]. As shown in Figure 6 and Figure 7, compared with the characteristic variables selected by SPA, more variables are activated by the third convolutional layer in the 1D-CNN models. The activation function also plays a central role in the prediction performance of 1D-CNN models [11]. Convolution is a linear operation. The feature maps of the convolutional layer passed the activation function to implement the nonlinear transformation. Thus, 1D-CNN with the outperformance of extracting the linear and nonlinear features in the spectra [18,33].

In addition, for the SPA-PLS and SPA-LS-SVM models, although the variable selection can effectively improve the prediction performance, the SPA algorithm does not consider the effect of the variable combination. The variable combination also has a significant effect on the prediction performance [12]. However, few of the variable selection algorithms have solved this problem [39].

#### 3.5.2. Impacts of Spectra Preprocessing on Calibration Models

After different preprocess for raw spectra, PLS and LS-SVM models were established. As shown in Table 1, for the spectrophotometer experiment, the LS-SVM model based on SG (2,7) smoothing preprocess achieved the highest prediction accuracy. Under some preprocessing methods, the prediction results decrease to some degree. As shown in Table 2, for the grating spectrograph experiment, the LS-SVM model based on Z-score normalization preprocess showed the best prediction performance. The results show that different preprocessing methods have quite different effects on PLS and LS-SVM models. Thus, spectra preprocessing plays an important role in traditional calibration modeling. Appropriate preprocessing methods can improve the prediction performance, but misuse of preprocessing methods will decrease the prediction performance.

For 1D-CNN models, the only required preprocessing is normalization [14]. As shown in Figure 6 and Figure 7, the first convolutional layer can process the spectra automatically. For the water pH detection experiments, 1D-CNN acquired the highest prediction accuracy. Therefore, the results indicate that the 1D-CNN regression strategy can achieve excellent prediction performance without the need for complex spectra pretreatment [11].

#### 3.5.3. Impacts of Feature Selection on Calibration Models

As shown in Figure 8a, for the spectrophotometer experiment, the prediction performance of SPA-PLS model is better than that of the PLS model. Under the SPA-PLS model, the RMSEP is 1.0209 and the $R_{p}^{2}$ is 0.7539. Compared with the PLS model, the RMSEP is reduced by 10.30%, and the $R_{p}^{2}$ is increased by 8.59%. However, it is worth noting that, under the SPA-LS-SVM model, the prediction accuracy decreased to some degree. Compared with the LS-SVM model, the RMSEP is increased by 9.63%, and the $R_{p}^{2}$ is reduced by 6.74%. According to the principle of the SPA algorithm, the characteristic wavelength variables are selected based on linear regression. Therefore, using these characteristic wavelength variables to establish a nonlinear model may reduce the prediction accuracy.

As shown in Figure 8b, for the grating spectrograph experiment, both the SPA-PLS and SPA-LS-SVM models acquired good prediction accuracy. Under the SPA-PLS model, the RMSEP is 0.5737, and the $R_{p}^{2}$ is 0.9145. Compared with PLS model, the RMSEP is reduced by 49.52%, and the $R_{p}^{2}$ is increased by 37.60%. Under the SPA-LS-SVM model, the RMSEP is 0.5211, and the $R_{p}^{2}$ is 0.9249. Compared with LS-SVM model, the RMSEP is reduced by 49.05%, and the $R_{p}^{2}$ is increased by 30.12%. The results further indicated that variable selection could effectively extract the informative variables and eliminate the noise and uninformative variables from the original spectra [18]. There are five characteristic variables located at the wavelength near 970 nm, which is related to the third overtones of O-H bonds in water [19]. The result indicates that the O-H bond in water is helpful to water pH prediction.

For 1D-CNN models, as the results show in Figure 6 and Figure 7, the convolutional layers can automatically extract the informative variables from full spectra. Uninformative variables are almost not activated, and information variables are highly activated [13]. Compared with the characteristic wavelength variables selected by SPA, the convolutional layers extract more hidden features of spectra. For both the spectrophotometer experiment and grating spectrograph experiment, 1D-CNN models based on full spectra acquired the best prediction accuracy. The results indicate that 1D-CNN can effectively extract the spectra features to build the relationship between spectra and water pH value.

#### 3.5.4. Discussion of Calculation Rapidity

This subsection discussed the calculation rapidity in the prediction process of different calibration models. The original spectra of the prediction set were inputted into the trained calibration models to calculate the prediction results. All calculations were repeated five times, and the statistical results of the calculation time are shown in Table 6.

As shown in Table 6, for the spectrophotometer experiment, 1D-CNN is faster than LS-SVM and SPA-LS-SVM in the prediction process. Although PLS and SPA-PLS are faster than 1D-CNN, PLS and SPA-PLS are not as accurate as 1D-CNN. For the grating spectrograph experiment, 1D-CNN is faster than SPA-PLS and SPA-LS-SVM. The PLS and LS-SVM are faster than 1D-CNN, but the prediction performance of 1D-CNN is better than PLS and LS-SVM. That is because 1D-CNN does not need the complex spectra preprocessing and variable selection, and 1D-CNN can effectively extract the linear and nonlinear features from the spectra. Therefore, 1D-CNN provides a better balance of prediction accuracy and calculation rapidity.

For the PLS and LS-SVM methods, in order to build a high-accuracy calibration model, a lot of experiments are needed to find the best preprocessing and variable selection methods. The spectra preprocessing and variable selection are repetitive and time-consuming. On the contrary, 1D-CNN only required preprocessing is normalization. Therefore, 1D-CNN could improve the convenience of modeling.

## 4. Conclusions

This study proposed a novel calibration regression strategy for distilled water pH detection using Vis-NIR spectroscopy, a 1D-CNN regression architecture with three convolutional layers constructed to optimize the prediction performance. Two groups of Vis-NIR spectral analysis experiments of water pH detection are employed to evaluate the performance of 1D-CNN. Two traditional multiple regression algorithms (PLS and LS-SVM) are introduced for comparison analysis with 1D-CNN. The conclusions are described as follows.

(1) The prediction performance of 1D-CNN based on full spectra is better than the traditional linear (PLS) and nonlinear (LS-SVM) approaches using full spectra and characteristic wavelength variables. For the spectrophotometer experiment, the RMSEP is 0.7925 and the $R_{p}^{2}$ is 0.8515. For the grating spectrograph experiment, the RMSEP is 0.5128 and the $R_{p}^{2}$ is 0.9273.
(2) (By visualizing the characteristic map through three convolution layers, we can understand how the convolution network converts one-dimensional spectral data into prediction results. The first convolutional layer acts for spectra pretreatment and learns the shape feature of input spectra. The second convolutional layer extracts the hidden features in the spectra. The third convolutional layer stably enhances the activations of the feature spectra peaks.
(3) 1D-CNN could effectively extract the spectra features. The number of activation variables of 1D-CNN is more than the feature variables selected by SPA, and the prediction accuracy of 1D-CNN is higher than that of SPA-PLS and SPA-LS-SVM for both experiments.
(4) 1D-CNN could improve the convenience of modeling. Compared with the traditional regression methods, 1D-CNN modeling only require preprocessing is normalization. 1D-CNN does not need complex spectra pretreatment and variable selection, which ensures the calculation rapidity of 1D-CNN.

This study indicates the 1D-CNN regression method is an alternative quantitative calibration technology for water pH detection using Vis-NIR spectroscopy. The future direction of our work is to explore the feasibility of the 1D-CNN regression method for other water quality parameters (such as chemical oxygen demand) detection and to develop a calibration transfer approach of water pH based on 1D-CNN.

## Figures and Tables

**Table 1:** Prediction parameters of PLS and LS-SVM with different preprocessing for the spectrophotometer experiment.

Model	Preprocessing	nPCs	*γ*	*δ* ^2^	Calibration Set		Prediction Set	
					RMSEC	$R_{c}^{2}$	RMSEP	$R_{p}^{2}$
PLS	Raw	9	-	-	0.3986	0.9621	1.1381	0.6942
	Smoothing	6	-	-	1.0119	0.7557	1.3038	0.5986
	SNV	3	-	-	1.6956	0.3141	1.8039	0.2318
	Z-score	5	-	-	1.1286	0.6961	1.1711	0.6762
LS-SVM	Raw	-	77,838.29	26,573.41	0.8957	0.8086	1.0295	0.7495
	Smoothing	-	85,781.59	29,349.56	0.9332	0.7923	1.0290	0.7498
	SNV	-	22,520.66	79,010.55	0.7688	0.8590	1.6613	0.3478
	Z-score	-	54,293.14	14,798.45	0.8296	0.8358	1.2398	0.6368

**Table 2:** Prediction parameters of PLS and LS-SVM with different preprocessing for the grating spectrograph experiment.

Model	Preprocessing	nPCs	*γ*	*δ* ^2^	Calibration Set		Prediction Set	
					RMSEC	$R_{c}^{2}$	RMSEP	$R_{p}^{2}$
PLS	Raw	8	-	-	0.0424	0.9995	1.1496	0.6569
	Smoothing	8	-	-	0.0754	0.9985	1.1366	0.6646
	SNV	6	-	-	0.1354	0.9954	1.2530	0.5924
	Z-score	6	-	-	0.2187	0.9882	1.2879	0.5694
LS-SVM	Raw	-	29,195.21	3095.09	0.0022	0.9999	1.1991	0.6025
	Smoothing	-	92,829.47	99,301.32	0.0293	0.9998	1.2294	0.5821
	SNV	-	89,171.26	1500.91	0.0001	0.9999	1.3533	0.4936
	Z-score	-	35,097.78	3077.16	0.0018	0.9999	1.0228	0.7108

**Table 3:** Prediction parameters of SPA-PLS and SPA-LS-SVM based on characteristic wavelength variables for the spectrophotometer experiment.

Model	nPCs	*γ*	*δ* ^2^	Calibration Set		Prediction Set	
				RMSEC	$R_{c}^{2}$	RMSEP	$R_{p}^{2}$
SPA-PLS	12	-	-	0.8760	0.9169	1.0209	0.7539
SPA-LS-SVM	-	98,472.83	2024.81	1.0019	0.7605	1.1286	0.6990

**Table 4:** Prediction parameters of SPA-PLS and SPA-LS-SVM based on characteristic wavelength variables for the grating spectrograph experiment.

Model	nPCs	*γ*	*δ* ^2^	Calibration Set		Prediction Set	
				RMSEC	$R_{c}^{2}$	RMSEP	$R_{p}^{2}$
SPA-PLS	8	-	-	0.1549	0.9941	0.5737	0.9145
SPA-LS-SVM	-	73,016.22	6037.97	0.0782	0.9985	0.5211	0.9249

**Table 5:** Prediction parameters of 1D-CNN models for the spectrophotometer experiment and grating spectrograph experiment.

Experiment	Calibration Set		Prediction Set	
	RMSEC	$R_{c}^{2}$	RMSEP	$R_{p}^{2}$
Spectrophotometer	0.7478	0.8715	0.7925	0.8515
Grating spectrograph	0.1337	0.9953	0.5128	0.9273

**Table 6:** Calculation time in the prediction process of different calibration models *.

Experiment	PLS (s)		LS-SVM (s)		SPA-PLS (s)		SPA-LS-SVM (s)		1D-CNN (s)	
	Mean	Std.	Mean	Std.	Mean	Std.	Mean	Std.	Mean	Std.
Spectrophotometer	0.0014	0.0005	0.0065	0.0004	0.0017	0.0002	0.0081	0.0009	0.0024	0.0005
Grating spectrograph	0.0068	0.0008	0.0065	0.0012	0.0186	0.0008	0.0232	0.0009	0.0082	0.0011

* All calculations were performed on a PC with an Intel^®^ Core^™^ i5-9500 CPU (3.0 GHz) and 8.0 GB RAM, running a Windows 11 operating system.

## Data Availability

The data presented in this study are available on request from the corresponding author. The data are not publicly available due to it is being used to apply for project.
